# The Incidental Finding of Trichilemmal Cyst in a Patient With Acute Kidney Injury

**DOI:** 10.7759/cureus.64657

**Published:** 2024-07-16

**Authors:** Vincent Lee, Sahar S Abdelmoneim, Rafael Sanchez-Dopazo, Miguel Huayta, Sohair Angly, Odalys Frontela

**Affiliations:** 1 Medicine, Nova Southeastern University Dr. Kiran C. Patel College of Osteopathic Medicine, Fort Lauderdale, USA; 2 Internal Medicine, Larkin Community Hospital Palm Springs Campus, Miami, USA; 3 General Internal Medicine/Cardiovascular Medicine, Assiut University Hospital, Assiut, EGY; 4 Medicine, Universidad Peruana de Ciencias Aplicadas, Lima, PER

**Keywords:** proliferating trichilemmal tumour, malignant proliferating trichilemmal tumours, trichilemmal cysts, malignant proliferating trichilemmal tumour, proliferating trichilemmal cysts, malignant proliferating trichilemmal cyst, trichilemmal carcinoma, proliferating trichilemmal tumor, trichilemmal cyst

## Abstract

This case highlights the importance of thorough clinical examinations from head to toe and the early diagnosis of trichilemmal cysts. We present a case of an incidentally discovered trichilemmal cyst in a 72-year-old patient who presented with acute kidney injury secondary to a urinary tract infection. In rare instances, these cysts can transform into malignant lesions. Therefore, clinicians should be aware of the potential for malignancy when diagnosing and managing trichilemmal cysts.

## Introduction

Benign skin lesions known as trichilemmal or pilar cysts arise from the outer sheath around hair roots. They usually appear as painless, slowly expanding nodules with a cystic, fluctuant consistency. Most common in females, these cysts typically present around the mean age of 65 and affect 5-10% of the population. Ninety percent of trichilemmal cysts are found on the scalp [1]. To distinguish these lesions from those related to the meninges or central nervous system, a workup including radiography, CT scans, and MRI may be performed. Surgical excision is the only definitive treatment, with the diagnosis confirmed by histopathologic analysis [2]. Here, we present a case of an incidentally discovered trichilemmal cyst in a 72-year-old patient who presented with acute kidney injury secondary to a urinary tract infection. The patient was informed that data concerning the case would be submitted for publication, and he provided informed consent.

## Case presentation

**Figure 1:**
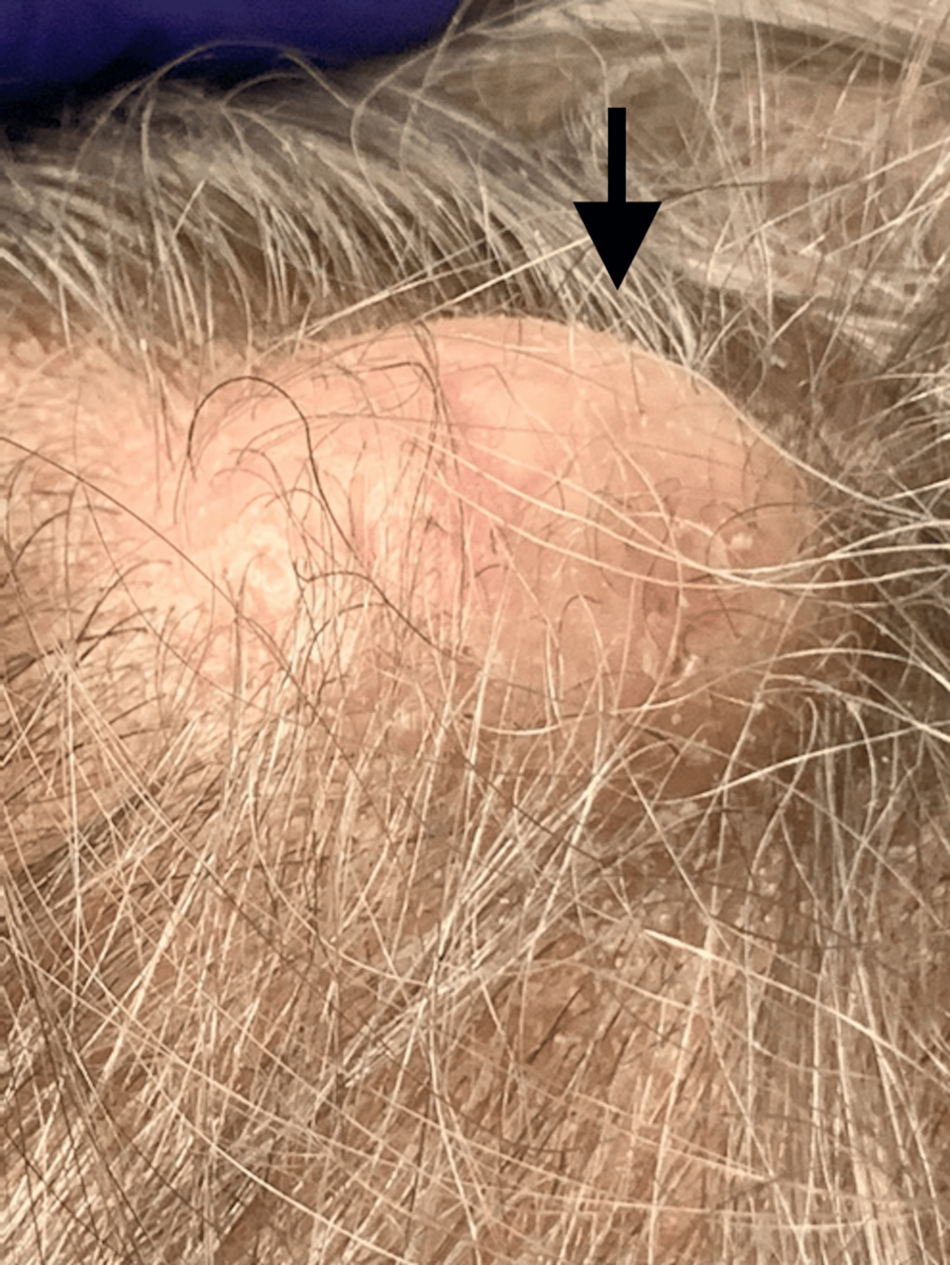
Trichilemmal cyst of the scalp

**Figure 2:**
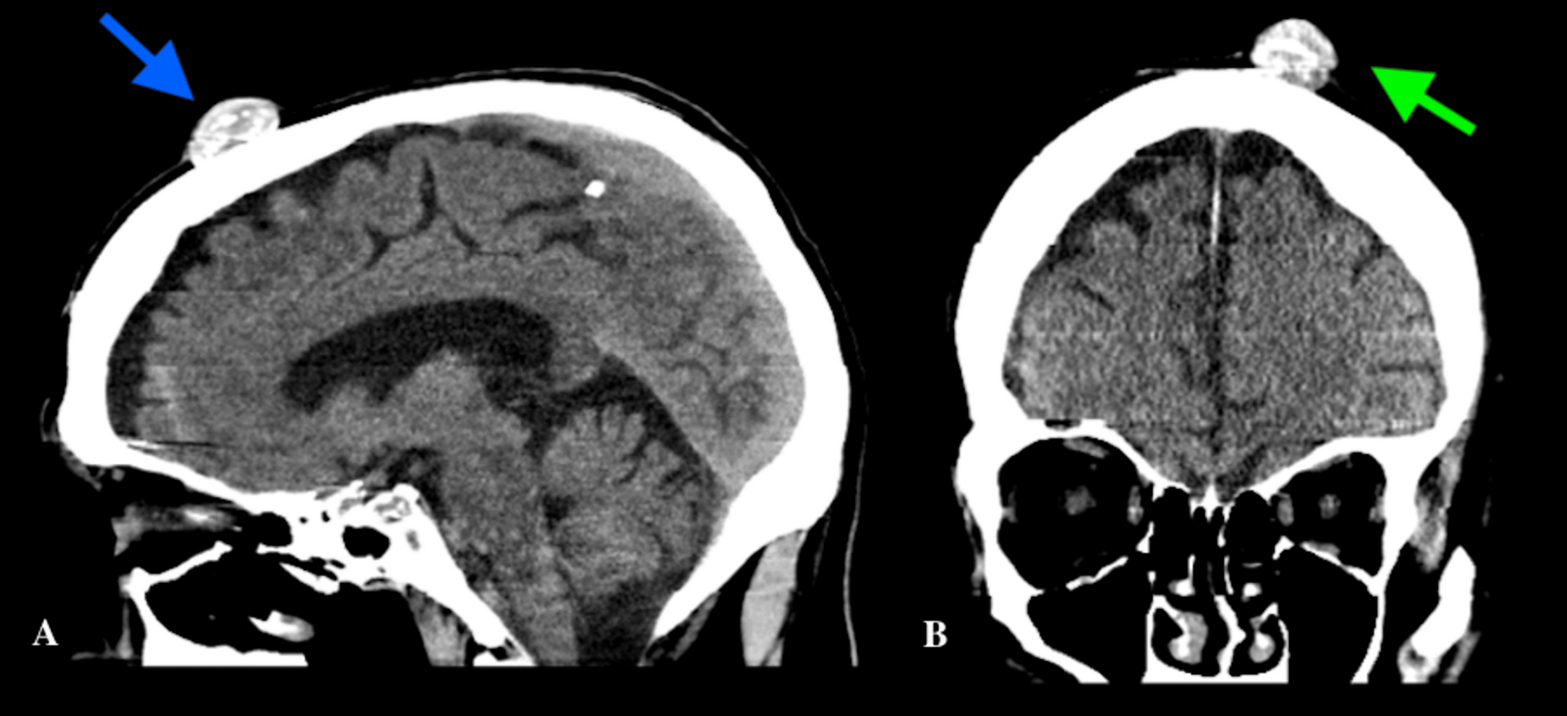
CT head of trichilemmal cyst (a) Sagittal CT view depicting mass lesion (blue arrow) and (b) coronal CT view displaying the same tissue lesion (green arrow)

We report a case of a 72-year-old Caucasian male who presented to the emergency department with a urinary tract infection, acute kidney injury, and hyperkalemia. The patient had a medical history of congestive heart failure, benign prostatic hyperplasia, major depressive disorder, hyperlipidemia, and hypertension. The patient’s home medications included acetaminophen, amlodipine, atorvastatin, clobetasol 0.05% ointment, escitalopram, ferrous sulfate, gabapentin, and tamsulosin. On evaluation, he was alert and oriented to person, time, and place. The patient denied fevers, chills, chest pain, palpitations, abdominal pain, vomiting, diarrhea, nausea, dysuria, and urinary frequency. However, he was a poor historian and reported bilateral lower extremity pain without providing further information. On admission, his vital signs were: blood pressure of 136/63 mmHg, pulse of 43 beats per minute, respiratory rate of 13 breaths per minute, afebrile, and oxygen saturation of 97% on room air. Abnormal results (reference range) of laboratory tests at presentation were: sodium, 123 mEq/L (135-145); potassium, 7.1 mEq/L (3.5-5.0); blood urea nitrogen, 160 mg/dL (8-20); creatinine, 15 mg/dL (0.6-1.11); brain natriuretic peptide, 16,000 pg/mL (<100); glomerular filtration rate, 3 mL/min (90-120); and white blood cells, 16,300 cells/μl (4,500-11,000). His significant urinalysis results were: white blood cells, 80-100 cells/hpf (0-2); red blood cells, 80-100 cells/hpf (0-3); and many bacteria (none). On physical examination, his lungs were clear to auscultation bilaterally, and his heart sounds were normal with a regular rate and rhythm. We found an incidental smooth, mobile, firm, and well-circumscribed nodule on the scalp (Figure 1). The surface appeared normal with no discoloration, scarring, punctum, or ulceration. The patient stated that the mass had never been painful, infected, or bleeding. A CT scan revealed a soft tissue lesion measuring 2.3 cm in diameter and 1.3 cm in size, overlying the left frontal bone with areas of calcification (Figure 2). Differential diagnoses include epidermoid cysts, dermoid cysts, and lipoma. Epidermoid cysts often have a central punctum and are lined by stratified squamous epithelium with a granular layer, distinguishing them from trichilemmal cysts, which lack a granular layer. Dermoid cysts contain ectodermal structures like hair follicles and sebaceous glands, which are absent in trichilemmal cysts. Lipomas are soft and composed of mature adipocytes, differing from the firm, keratin-filled trichilemmal cysts. The patient underwent emergent hemodialysis and was started on cefepime and calcium gluconate for the initial management of hyperkalemia. His clinical condition improved, and the hospital stay was uneventful. He was discharged back to his nursing facility on the eighth day. The patient was referred for a surgical consult as an outpatient. While this incidental finding of a trichilemmal cyst on the scalp was unrelated to the initial presentation, it is important to recognize such findings clinically.

## Discussion

This case report emphasizes the value of a comprehensive clinical examination that covers the entire body, including the differential diagnosis of scalp cysts, which can include trichilemmal cysts. Trichilemmal cysts have several distinguishing features, including solid or slightly spongy, spherical, movable nodules that grow slowly beneath the skin, usually on the scalp, though they can occur elsewhere in the body [2]. Their content is mostly keratin, a protein found in hair and nails. Trichilemmal cysts are typically managed by addressing the noticeable nodules, tenderness, erythema, and potential rupture while also preventing infection, chronic inflammation, rupture and scarring, cosmetic concerns, and recurrence. Additionally, surgical removal may become necessary [3]. Trichilemmal cyst imaging has been investigated using CT and MRI [1]. On CT scans, a trichilemmal cyst appears as a well-enhancing wall of variable thickness with sporadic speckled calcifications, but usually with no evidence of extracapsular spread. While trichilemmal cysts often grow benignly over several months to years, there have been reports of malignant transformation leading to local invasion or metastasis into a proliferating trichilemmal cyst (PTC) [4]. Though new evidence suggests that malignancy may occur in about 30% of PTCs, the actual incidence of malignant transformation remains uncertain. Cases of lung metastasis and intracranial involvement have been documented, indicating the possibility of a fatal course for PTC [4]. Moreover, there have been reports of rapidly growing, painful trichilemmal cysts progressing to trichilemmal carcinoma [5]. The risk of transformation into a malignant tumor has been shown to be higher than previously reported. In a meta-analysis by Satyaprakash et al. [2], local recurrence was found to be 3.7% after wide local excision. To date, several case reports have documented instances of trichilemmal cysts undergoing malignant transformation (Table 1).

**Table 1:** Previous reports of malignant transformation in trichilemmal cyst

Study	Age/sex	Initial findings	Transformation	Metastasis
Kruse-Lösler et al. [6]	77/female	Giant proliferating trichilemmal cyst of the scalp	Trichilemmal carcinoma	Retroclavicular lymph node metastases
Fernandez [7]	86/male	A painless, pea-sized pilar cyst located on the left angle of the jaw	Malignant proliferating trichilemmal tumor	Left jugular lymph node
Khaled et al. [8]	57/female	Scalp lesion with multiple nodules exhibiting spontaneous and profuse bleeding	Malignant proliferating trichilemmal cyst	None
Lobo et al. [4]	29/male	Scalp swelling	Malignant proliferating trichilemmal tumor	Multiple nodules in lungs bilaterally
ElBenaye et al. [9]	70/female	Progressive enlargement of an occipital cyst over the span of six years	Malignant proliferating trichilemmal tumor	Tumor recurrence with regional metastases and intracerebral invasion after four months
Dboush et al. [10]	45/female	Right axillary mass of six-month duration	Malignant adnexal skin tumor with foci of trichilemmal-type keratinisation	None
Benslimane et al. [11]	Unknown	Changes in a firm nodular scalp lesion, characterized by budding, ulceration, and infiltration. Brain scan revealed bone lysis of the outer table of the parietal vault.	Malignant proliferating trichilemmal cysts	Tumor recurrence in two months and death a few months afterward

## Conclusions

This case report underscores the importance of conducting comprehensive clinical examinations to identify incidental findings of scalp cysts, such as trichilemmal cysts. Despite their typically benign nature, trichilemmal cysts can occasionally undergo malignant transformation, necessitating timely recognition and appropriate management. Our patient, initially presenting with acute kidney injury secondary to a urinary tract infection, was incidentally found to have a trichilemmal cyst on the scalp. While unrelated to the initial presentation, this finding emphasizes the need for clinicians to remain vigilant for unexpected pathologies during routine evaluations. By recognizing and addressing such incidental findings, clinicians can ensure optimal patient care and outcomes.

## References

[1] Kawaguchi M, Kato H, Suzui N (2021). Imaging findings of trichilemmal cyst and proliferating trichilemmal tumour. Neuroradiol J.

[2] Satyaprakash AK, Sheehan DJ, Sangüeza OP (2007). Proliferating trichilemmal tumors: a review of the literature. Dermatol Surg.

[3] Kiel CM, Homøe P (2021). Giant, bleeding, and ulcerating proliferating trichilemmal cyst, with delayed treatment due to coronavirus outbreak: a case report and review of the literature. Front Surg.

[4] Lobo L, Amonkar AD, Dontamsetty VV (2016). Malignant proliferating trichilemmal tumour of the scalp with intra-cranial extension and lung metastasis-a case report. Indian J Surg.

[5] Short E, O'Shea A, Mukkanna K, Patel G, Docjinov S, May K (2019). Case Report: a rapidly growing cyst on the scalp. F1000Res.

[6] Kruse-Lösler B, Kleinheinz J, Werkmeister R, Piffko J, Metze D, Joos U (1998). [Gigantic proliferating trichilemmal cyst of the scalp with central carcinoma and lymph node metastasis]. Mund Kiefer Gesichtschir.

[7] Fernandez SH (1999). Malignant proliferating trichilemmal tumour: a case report. Malays J Pathol.

[8] Khaled A, Kourda M, Fazaa B, Kourda J, Ben Jilani S, Kamoun MR, Zermani R (2011). Malignant proliferating trichilemmal cyst of the scalp: histological aspects and nosology. Pathologica.

[9] ElBenaye J, Elkhachine Y, Sakkah A, Sinaa M, Moumine M, Jakar A, Elhaouri M (2018). [Malignant proliferating trichilemmal cyst of the scalp: a case report]. Ann Chir Plast Esthet.

[10] Dboush HG, Al-Doud MA, Shannaq RY, Abudarweesh IS, Jabali EH, Alabbadi AS (2021). Trichilemmal carcinoma of the axilla with regional lymph nodes metastasis: A case report. Int J Surg Case Rep.

[11] Benslimane Kamal I, Hali F, Marnissi F, Chiheb S (2022). The proliferating and malignant proliferating trichilemmal cyst: an anatomo-clinical study of three cases. Skin Appendage Disord.

